# Effects of Elevated CO_2_ and Temperature on Yield and Fruit Quality of Strawberry (*Fragaria* × *ananassa* Duch.) at Two Levels of Nitrogen Application

**DOI:** 10.1371/journal.pone.0041000

**Published:** 2012-07-24

**Authors:** Peng Sun, Nitin Mantri, Heqiang Lou, Ya Hu, Dan Sun, Yueqing Zhu, Tingting Dong, Hongfei Lu

**Affiliations:** 1 College of Chemistry and Life Science, Zhejiang Normal University, Jinhua, China; 2 School of Applied Sciences, Health Innovations Research Institute, RMIT University, Melbourne, Victoria, Australia; University of Illinois, United States of America

## Abstract

We investigated if elevated CO_2_ could alleviate the negative effect of high temperature on fruit yield of strawberry (*Fragaria* **×** *ananassa* Duch. cv. Toyonoka) at different levels of nitrogen and also tested the combined effects of CO_2_, temperature and nitrogen on fruit quality of plants cultivated in controlled growth chambers. Results show that elevated CO_2_ and high temperature caused a further 12% and 35% decrease in fruit yield at low and high nitrogen, respectively. The fewer inflorescences and smaller umbel size during flower induction caused the reduction of fruit yield at elevated CO_2_ and high temperature. Interestingly, nitrogen application has no beneficial effect on fruit yield, and this may be because of decreased sucrose export to the shoot apical meristem at floral transition. Moreover, elevated CO_2_ increased the levels of dry matter-content, fructose, glucose, total sugar and sweetness index per dry matter, but decreased fruit nitrogen content, total antioxidant capacity and all antioxidant compounds per dry matter in strawberry fruit. The reduction of fruit nitrogen content and antioxidant activity was mainly caused by the dilution effect of accumulated non-structural carbohydrates sourced from the increased net photosynthetic rate at elevated CO_2_. Thus, the quality of strawberry fruit would increase because of the increased sweetness and the similar amount of fruit nitrogen content, antioxidant activity per fresh matter at elevated CO_2_. Overall, we found that elevated CO_2_ improved the production of strawberry (including yield and quality) at low temperature, but decreased it at high temperature. The dramatic fluctuation in strawberry yield between low and high temperature at elevated CO_2_ implies that more attention should be paid to the process of flower induction under climate change, especially in fruits that require winter chilling for reproductive growth.

## Introduction

The Intergovernmental Panel on Climate Change (IPCC) reported that rising temperatures, drought, floods, desertification and weather extremes will severely affect agricultural production, especially in developing countries [1]. The CO_2_ concentration near the ground level has risen from 280 mmol mol^−1^ in the pre-industrial times to the present 390 mmol mol^−1^ [1]. At the present rate of emission, CO_2_ concentration is projected to be in the range of 500–1000 mmol mol^−1^ by the end of this century, which will potentially increase global temperature by 1.8–5.8°C [1]. Higher temperature individually or along with the ongoing global increase of atmospheric CO_2_ could affect various physiological and morphological traits of crops that subsequently influence crop growth and final yield. As estimated by Xiong et al. [2], in China, without the CO_2_ fertilization effect, grain yields of rice, wheat and maize would fall consistently if temperature rises by 2.5°C; even taking the CO_2_ fertilization effect into account, the yield reductions of these crops would still occur if temperature rises by 3.9°C. Therefore, it’s necessary and important to conduct research focusing on the combined effect of elevated CO_2_ and increased temperature on crop yield.

Strawberry (*Fragaria* × *ananassa* Duch.) is one of the most important fruit crops that is widely planted in North America, Mediterranean Europe, Southwest Asia, and Australia [3]. Shortened photoperiod and low temperature are known to induce flower formation for June-bearing strawberries [4]. Kumakura and Shishido [5] suggested that maximum strawberry yields are associated with a narrow range of temperatures between 15 and 20°C. The yield is reduced when the day temperature exceeded 25°C, even if the diurnal mean temperature is maintained below 20°C. Therefore, in the event of increased temperatures due to global warming, strawberry production would be severely affected. Currently, there is little knowledge of the combined effects of high temperature and elevated CO_2_ on strawberries or other crops, although published data suggest that such interaction is critical. Chen et al. [6] reported that elevated CO_2_ levels greatly improved yield and fruit quality of strawberry by increasing the total fruit number per plant, average fruit fresh weight, dry matter content, fruit total sugars and sugar/acid ratio. On the contrary, combined effect of elevated CO_2_ and temperature on other C_3_ crops such as rice, soybean, dry bean, peanut, cowpea, wheat and cotton cultivated in different growth conditions, including growth chambers, open-top chambers and plastic tunnels, showed no beneficial effect on yield [7]–[13]. However, strawberries require much lower temperature than these crops, and it is important to test whether elevated CO_2_ will ameliorate the negative effects of the increased temperature on its reproductive development.

Nitrogen is one of the most important resources limiting plant growth and seed production in natural and agricultural ecosystems [14]. An increase in carbon availability due to elevated CO_2_ may enhance nitrogen limitation, leading to a reduction in plant nitrogen concentration [15]. Studies on spring wheat and rice suggested that under elevated CO_2_ concentration, nitrogen fertilization had important influence on the maintenance and continuing increase of crop yield [16]–[18]. The deeper and larger root system with nitrogen fertilization, which is of benefit to the use of soil moisture and nutrient, is thought to be the reason of continuing increase of crop yield [16]. Deng and Woodward [19] reported high CO_2_ increased the strawberry fruit yield by 42% at high nitrogen supply and 17% at low nitrogen supply through an increase in flower and fruit number of individual plants. However, they did not analyze the effect of high temperature, elevated CO_2_ and nitrogen supply on strawberry fruit production and quality.

Strawberries are a good source of natural antioxidants [20]. In addition to the usual nutrients, such as vitamins and minerals, strawberries are also rich in anthocyanins, flavonoids, and phenolic acids [20]. Strawberries have shown a remarkably high scavenging activity toward chemically generated radicals, thus making them effective in inhibiting oxidation of human low-density lipoproteins [20]. At elevated CO_2_, decrease, no change, and an increase in fruit antioxidant activity have been reported [21]–[24]. Levine and Paré [24] showed in scallions that both, total phenol and total antioxidant activity decrease under elevated CO_2_. They suggested that besides species differences, in the absence of stress, plant grew with minimum investment in antioxidant compounds to maintain a basal defense level under elevated CO_2_. Contrastingly, the increase of fruit antioxidant activity may stem from the reduction of fruit nitrogen concentration induced by the elevated CO_2_. As a ‘physiological trade-off’, the amount of secondary metabolites like phenolics increases at low nitrogen to maintain the growth-differentiation balance (GDB) framework [25]. Further, the antioxidant activity of plant tissues also increases as reactive oxygen species (ROS) that are involved in the signaling and perception of nitrogen deficiency increase [26].

Due to the prediction of climate change, a number of studies have examined the effects of rising CO_2_ and/or temperature on yield characteristics, notably quantity and nutrition of food crops [7]–[13], [16]–[18]. However, almost nothing is known regarding the concurrent interaction of CO_2_, temperature and nutrition (e.g. N) on reproductive biology of fruit crops. This is the first study to undertake an assessment of these potential interactions. Further, unlike the crops that have been studied, the temperature requirement for strawberry cultivation is quite low and the response of strawberry to the increased temperature may therefore be different to other crops. Therefore, we assessed the combined effects of CO_2_ concentration, air temperature and nitrogen application on the fruit yield and quality of strawberry. Firstly, we examined the fruit yield under these abiotic factors, and tested whether elevated CO_2_ can modify the response of fruit yield to elevated temperature. The effects of nitrogen supply on the response to fruit yield at elevated CO_2_ concentration and temperature were also studied. Secondly, we examined the combined effects of CO_2_ concentration, temperature and nitrogen supply on fruit quality such as carbohydrate accumulation, nitrogen content and antioxidant levels.

## Results

### Variation in Fruit Weight and Yield

**Figure 1:**
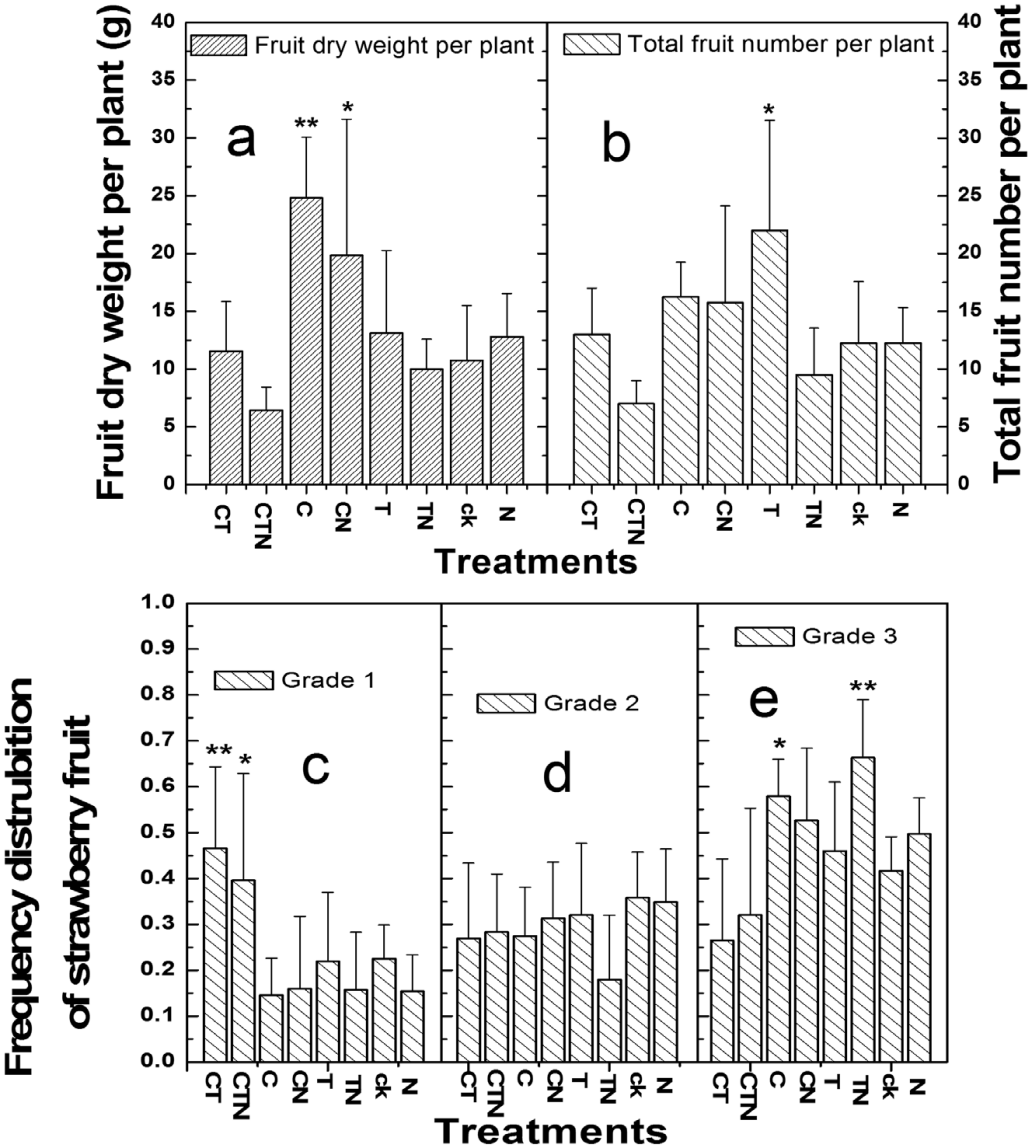
Total fruit dry weight (a), total fruit number (b) and fruit grades (c, d, e) of strawberry plants cultivated under different conditions (mean ± SD, n = 4). The berries were graded in three size classes depending on fruit dry weight (FDW; FDW <0.4 g, grade 1; 0.4≤ FDW ≤0.7 g, grade 2; FDW >0.7 g, grade 3). The frequency distribution of grade 1, 2 and 3 is showed in figures c, d and e, respectively. Bars indicate standard deviation, while ^*^ and ^**^ indicate significant differences at *P*<0.05 and 0.01, respectively.

The abbreviations for the combined treatments of different CO_2_ concentrations, temperatures and nitrogen concentrations reported below are explained in Table 1.Elevated CO_2_ increased fruit yield (viz. total fruit dry weight per plant) at low temperature, but deceased it at high temperature, when compared to the corresponding treatments in ambient CO_2_ (Figure 1a). The greatest fruit yield was in high CO_2_, low temperature and low nitrogen treatment (C), while the least was in high CO_2_, high temperature and high nitrogen treatment (CTN). The plants grown at low nitrogen concentration had greater yield than those grown at high nitrogen concentration, except for plants grown in low CO_2_, low temperature and low nitrogen treatment (ck). Similarly, elevated CO_2_ increased fruit number per plant (FN) at low temperature, but decreased it at high temperature, when compared to the corresponding treatments in ambient CO_2_ (Figure 1b). High nitrogen decreased FN at high temperature, but had no effect on FN at low temperature. FN in high CO_2_, high temperature and low nitrogen treatment (CT) was 1.86 times greater than in CTN treatment. Meanwhile, FN was 2.32 times greater in low CO_2_, high temperature and low nitrogen treatment (T) than in low CO_2_, high temperature and high nitrogen treatment (TN). The greatest FN was found in T treatment, which was significantly greater than in other treatments.

**Table 1:** Treatments performed in controlled growth chambers that were applied to strawberry plants for nearly 6 months^a^.

Description	Denoted
Increase in CO_2_ (720 ppm)	C
Increase in temperature (25°C/20°C; day temperature/night temperature)	T
Increase in nitrogen fertilizer input (50 ml of 0.1% NH_4_NO_3_ twice a week per plant )	N
Increase in CO_2_, temperature and nitrogen input	CTN
Increase in CO_2_ and temperature	CT
Increase in CO_2_ and nitrogen input	CN
Increase in temperature and nitrogen input	TN
Control (360 ppm×20°C/15°C×without nitrogen input)	ck

a Values in round brackets indicate the detailed factors designed in the experiment.

Fruit dry weight (FDW) varied significantly (P<0.0001) both between treatments and between individual plants, thus the healthy fruits were graded in three size classes (grade 1<0.4 g, 0.4≤ grade 2≤0.7 g, grade 3>0.7 g; Figure 1c, 1d and 1e). Frequency distribution (FD) of the fruits in these three grades varied among all treatments. Elevated CO_2_ increased FD in grade 1 at high temperature, but deceased it at low temperature, when compared to the corresponding treatments in ambient CO_2_ (Figure 1c). Contrastingly, elevated CO_2_ decreased FD in grade 3 at high temperature, but increased it at low temperature, when compared to the corresponding treatments in ambient CO_2_ (Figure 1e). High nitrogen decreased FD in grade 1, but increased it in grade 3. The relatively lower fruit number and the highest FD in grade 1 in CT and CTN treatments eventually decreased fruit yield, when compared with the corresponding treatment in ambient CO_2_. While, the highest fruit yield in C and high CO_2_, low temperature and high nitrogen (CN) treatments resulted from the lowest FD in grade 1, second greatest FD in grade 3 and second greatest FN.

**Figure 2:**
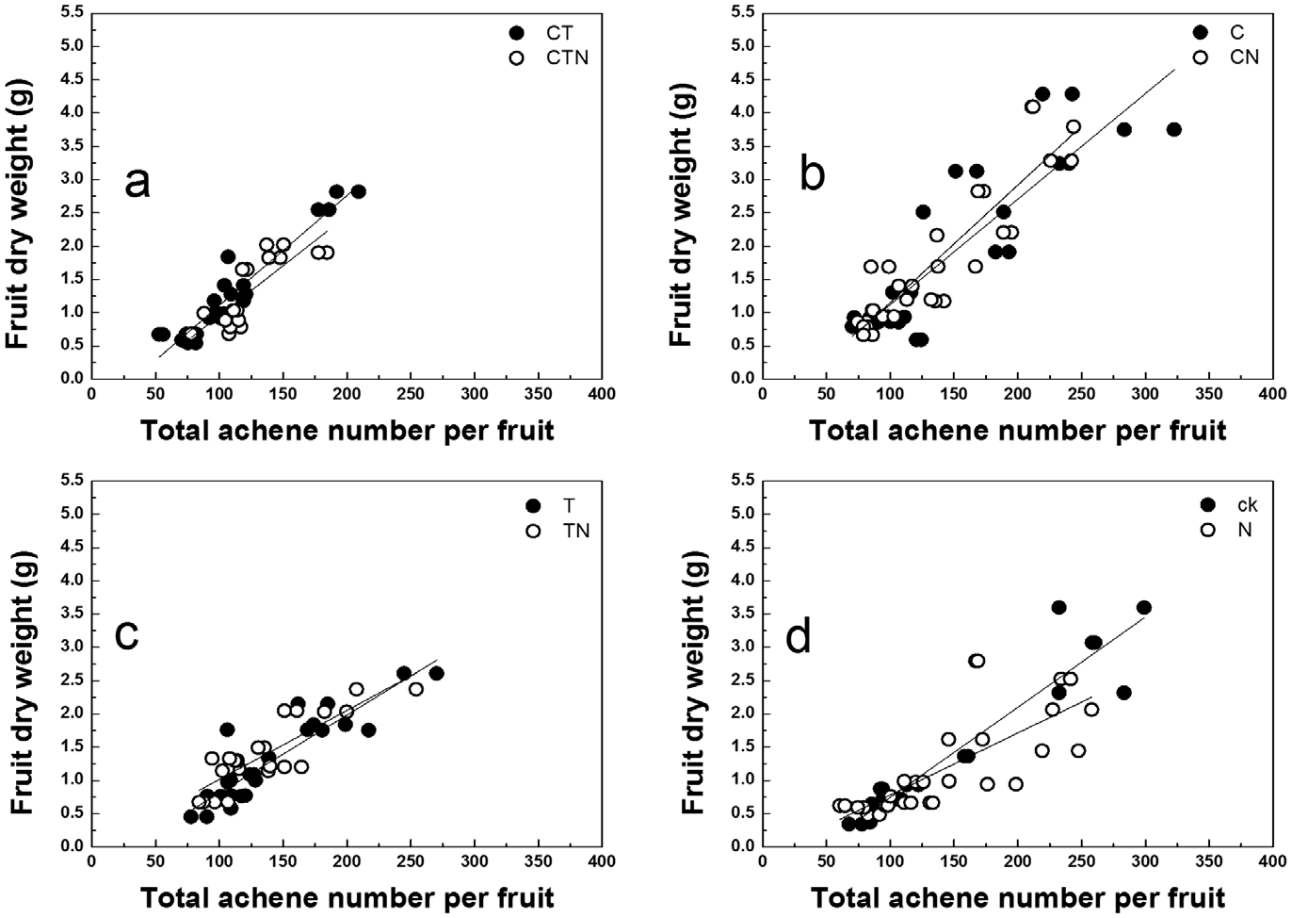
Correlations between fruit dry weight and total achene number (TAN) of strawberry fruits for plants grown in different conditions (a, b, c and d). The linear regression: y  =  a x + b.

**Figure 3:**
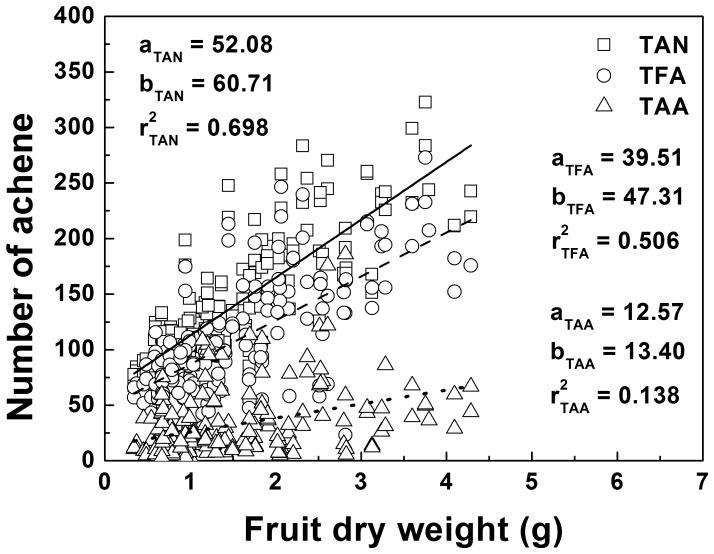
Correlations between pooled fruit dry weight and total achene number (TAN, open square), total number of fertilized achenes (TFA, open circle) and total number of aborted achenes (TAA, open triangle) of strawberry fruits for plants grown in different conditions. Regression lines: solid line, TAN; broken line, TFA; dotted line, TAA. Regression lines: y = a x + b.

A linear regression was performed to compare slopes of relationships between FDW and total achene number (TAN) on the surface of fruit (Figure 2a, 2b, 2c and 2d). The slopes of FDW versus TAN appeared greater for high CO_2_ treated plants than low CO_2_ treated plants, while the slopes decreased at high nitrogen when averaged over the other factors (Table 2). Pooling TAN across all treatments revealed that the correlation coefficient between TAN and FDW was significant (r^2^ = 0.698, P<0.001) and higher than the correlation coefficient between total fertilized achene number (TFA) and FDW (r^2^ = 0.506, P<0.001, Figure 3). However, the relationship between total aborted achene number (TAA) and FDW was very week (r^2^ = 0.138; Figure 3), and these different patterns suggested that the aborted achenes were not the major limitation to FDW.

**Table 2:** Correlations between fruit dry weight and total achene number (TAN) of strawberry fruits grown in different conditions^a^.

Treatments	a	b	r^2^
**CT**	0.0165	−0.539	0.913**
**CTN**	0.0150	−0.541	0.657**
**C**	0.0159	−0.463	0.771**
**CN**	0.0177	−0.617	0.815**
**T**	0.0117	−0.360	0.808**
**TN**	0.0104	−0.020	0.725**
**ck**	0.0136	−0.611	0.906**
**N**	0.0093	−0.148	0.563**

a the linear regression: y  =  a x + b, a-slope of linear regression, b-increment of linear regression, r-correlation coefficient.

** indicate P<0.01.

### Variation in Taste and Health-related Compounds

Compared to the corresponding treatments in ambient CO_2_, elevated CO_2_ decreased the antioxidant compounds and total antioxidant capacity (in simple terms, antioxidant activity) of strawberry fruit in both high-temperature and low-temperature treatments (Table 3). As expected, the response of antioxidant capacity to the CO_2_ and temperature treatments was altered by nitrogen application, which increased at elevated CO_2_ but decreased in ambient CO_2_ with increasing nitrogen supply (Table 3). CO_2_ and nitrogen both significantly affected the total antioxidant capacity and all antioxidant compounds in strawberry fruit (Table 4). Compared to the corresponding treatments in ambient CO_2_, anthocyanin (AC) content decreased 27% in CT treatment and 48% in C treatment, but decreased only 1% and 4% in CTN and CN treatments, respectively (Table 3). There were significant CO_2_-temperature-nitrogen (C**×**T**×**N), CO_2_-temperature (C**×**T), CO_2_-nitrogen (C**×**N), and temperature-nitrogen (T**×**N) interactions affecting AC (Table 4). The treatment effects on total phenolics (TP) closely matched that of AC. Strawberry fruits showed a 27% and 21% decline in TP levels in CT and C treatments, respectively, but decreased only 8% in CTN treatment and 10% in CN treatment, when compared to the corresponding treatments in ambient CO_2_ (Table 3). There were significant CO_2_ and nitrogen main effects, and significant C**×**T, C**×**N and T**×**N interactions (Table 4) affecting TP levels. Total flavonoid (TF) decreased 31% and 36% in CT and C treatments, respectively, but decreased only 13% in both CTN and CN treatments, when compared to the corresponding treatments in ambient CO_2_ (Table 3). Besides significant CO_2_, temperature and nitrogen main effects, all interactions also affected the TF levels under various treatments (Table 4). Total antioxidant capacity measured using the free radical 2, 2-diphenyl-1-picrylhydrazyl (DPPH) method decreased 28% in CT treatment and 20% in C treatment, but decreased 12% and 13% in CTN and CN treatments, respectively (Table 3). Comparatively, total antioxidant capacity measured using the 2, 2′-azino-bis (3-ethylbenzothiazoline-6-sulphonic acid) (ABTS) method, which closely matched DPPH, decreased approximately 19% and 18% in CT and C treatments, respectively, but decreased 12% in CTN treatment and 8% in CN treatment, when compared with the corresponding treatments in ambient CO_2_ (Table 3). There were significant C**×**T**×**N, C**×**T, and C**×**N interactions affecting DPPH levels (Table 4). Similarly, all interactions had significant effects on the levels of ABTS in different treatments (Table 4). Fruit nitrogen contents (FNC) at elevated CO_2_ were similar to the corresponding treatments in ambient CO_2_, except the one in C treatment (Table 3). Contrastingly, high temperature increased the levels of FNC among all treatments with only an exception of T treatment. Not surprisingly, nitrogen application significantly increased the levels of FNC. There were only significant nitrogen and temperature main effects on FNC level (Table 4).

**Table 3:** Effects of carbon dioxide, temperature and nitrogen treatments on anthocyanin (AC), total phenolic (TP), total flavonoid (TF), DPPH radical scavenging assay (DPPH), ABTS radical scavenging assay (ABTS) and fruit nitrogen content (FNC) of strawberry fruits^a^.

	CT	CTN	C	CN	T	TN	ck	N
**AC (mg g^−1^ DW)**	2.34±0.51^*^	2.66±0.49^*^	1.74±0.24	2.21±0.47^*^	3.19±0.89^**^	2.69±0.35^*^	3.35±0.89^**^	2.30±0.28^*^
**TP (mg g^−1^ DW)**	13.57±0.74	13.03±1.46	13.30±2.54	14.21±1.23	18.61±4.06^***^	14.18±2.10	16.88±3.76^**^	15.77±1.28^*^
**TF (mg g^−1^ DW)**	4.59±0.50	4.62±0.89	4.57±0.74	5.48±1.36^*^	6.62±1.98^**^	5.33±0.81^*^	7.15±0.82^***^	6.31±0.86^**^
**DPPH (µmol g^−1^ DW)**	76.85±5.41	79.87±17.47	84.04±11.06	83.87±7.01	106.97±35.0^**^	90.58±14.17	104.9±40.5^**^	96.66±8.83^*^
**ABTS (µmol g^−1^ DW)**	84.12±11.60	80.22±15.45	84.49±15.89	93.20±5.53	103.97±25.81^*^	90.81±8.45	103.08±33.8^*^	101.39±4.62^*^
**FNC (mg g^−1^ DW)**	13.72±0.88	16.71±2.42^*^	11.74±2.93	14.87±1.92^*^	13.68±3.65^*^	16.81±3.41^*^	14.96±2.59^*^	14.83±1.81^*^

a Data are expressed as mean ± SD, n = 12, while *, ** and *** indicate P<0.05, 0.01 and 0.001, respectively. Abbreviations are: DW- dry weight.

**Table 4:** MGLM analysis of treatment (CO_2_, temperature and nitrogen) main effects and their interactions on AC, TP, TF, DPPH, ABTS and FNC of strawberry fruits for plants cultivated at ambient (360 ppm) and elevated (720 ppm) CO_2_, high and low temperature, and high and low nitrogen^a^.

	Effects							MS error	Whole model R^2^
	CO_2_	Temperature	Nitrogen	CO_2_×Temp	CO_2_×N	Temp×N	3-Way		
**AC**	243.13^****^	0.01	58.45^****^	7.64^*^	44.95^****^	76.88^****^	5.89^*^	0.08	0.94
**TP**	45.13^****^	7.02^*^	43.93^****^	19.90^****^	118.58^****^	4.21	92.29^****^	0.05	0.92
**TF**	807.89^****^	230.04^****^	52.05^****^	52.87^****^	4.36^*^	83.18^****^	69.21^****^	0.20	0.98
**DPPH**	1660.9^****^	0.39	343.35^****^	7.41^*^	358.39^****^	0.72	22.13^***^	9.28	0.99
**ABTS**	487.69^****^	0.59	160.50^****^	33.94^****^	270.70^****^	40.68^****^	50.84^****^	18.34	0.97
**FNC**	2.31	4.55^*^	18.40^***^	2.16	2.16	2.15	2.55	6.83	0.22

a Data are expressed as F values, and *, **, ***, **** indicate P<0.05, 0.01, 0.001 and 0.0001, respectively. Abbreviations are: FW- fresh weight; Temp- Temperature; N- Nitrogen; 3-Way- CO_2_×Temperature**×**Nitroge.

The concentrations of three main sugars (viz., fructose, glucose and sucrose) were also determined for each treatment; fructose and glucose were quantitatively the most important in this study. The contents of fructose and glucose at elevated CO_2_ were almost 1.3 times (1.29 and 1.35 times, respectively) higher than in ambient CO_2_ regardless of temperature and nitrogen treatments (Table 5). There was no significant difference in sucrose concentration in different treatments. There was significant effect of CO_2_, temperature, and C**×**T, C**×**N and C**×**T**×**N interactions on fructose concentration (P<0.05), whilst only CO_2_, C**×**T and C**×**T**×**N interactions significantly affected glucose concentration in different treatments (Table 6). Total sugars per gram fresh weight (TSW) averagely increased 43% under elevated CO_2_ regardless of temperature and nitrogen treatments (Table 5), and CO_2_ had a significant effect on TSW when compared to other factors (Table 6). Despite differences in the sugar distribution among the treatments, the ranking of sweetness index (SI) was similar to the ranking of total sugars (per fresh weight) from 86.4 to 128.8 relative units. CO_2_ effect was significant as it resulted in a 49%, 38%, 45% and 36% increase in SI in CT, CTN, C and CN treatments, respectively (Table 5, 6), when compared to the corresponding treatments in ambient CO_2_.

**Table 5:** Effects of carbon dioxide, temperature and nitrogen treatments on fructose (Fru), glucose (Glu), sucrose (Suc), total sugars (TSW), sweetness index (SI) and dry matter-content (DMC) of strawberry fruits^a^.

	CT	CTN	C	CN	T	TN	ck	N
**Fru (mg g^−1^ DW)**	291.7±22.1^**^	301.7±54.9^**^	264.5±28.8^*^	301.9±41.4^**^	225.7±50.8	186.4±44.1	259.1±43.6^*^	229.0±43.6
**Glu (mg g^−1^ DW)**	299.2±34.0^**^	294.3±77.1^**^	252.8±36.7^*^	288.3±34.8^**^	204.0±54.6	180.5±63.4	238.3±27.6	216.3±54.4
**Suc (mg g^−1^ DW)**	73.75±25.01	51.38±59.21	86.24±58.50	86.14±65.34	107.63±84.93	108.08±45.61	41.57±57.32	49.52±66.65
**TSW (mg g^−1^ FW)**	80.26±15.91^*^	77.19±18.57^*^	79.29±7.84^*^	75.77±9.82^*^	53.54±10.90	57.14±10.85	53.67±10.62	54.80±15.24
**SI (mg g^−1^ FW)**	128.8±25.9^*^	126.1±29.4^*^	128.4±11.0^*^	121.8±15.9^*^	86.4±16.6	91.5±17.9	88.5±16.1	89.6±23.8
**DMC**	0.127±0.014^*^	0.132±0.017^*^	0.131±0.015^*^	0.114±0.010	0.099±0.019	0.115±0.004	0.098±0.013	0.100±0.072

a Data are expressed as mean ± SD, n = 12, while * and ** indicate P<0.05 and 0.01 respectively. Abbreviations are: DW- dry weight; FW- fresh weight.

**Table 6:** MGLM analysis of treatment (CO_2_, temperature and nitrogen) main effects and their interactions on Fru, Glu, Suc, TSW, SI and DWC of strawberry fruits for plants cultivated at ambient (360 ppm) and elevated (720 ppm) CO_2_, high and low temperature, and high and low nitrogen^a^.

	Effects							MS error	Whole model R^2^
	CO_2_	Temperature	Nitrogen	CO_2_×Temp	CO_2_×N	Temp×N	3-Way		
**Fru**	24.79^****^	4.58^*^	0.05	5.51^*^	4.38^*^	0.15	6.10^*^	1464.02	0.58
**Glu**	22.54^****^	1.94	0.05	5.37^*^	1.31	0.52	7.92^*^	2079.33	0.53
**Suc**	0.2	0.34	0.67	2.47	1.15	0.26	0.65	3391.86	−0.03
**TSW (FW)**	88.41^****^	3.29	0.47	1.02	0.15	0.05	0.37	120.90	0.64
**SI (FW)**	101.15^****^	2.92	0.52	1.68	0.03	0.08	0.29	277.05	0.67
**DMC**	22.93^****^	2.22	0.08	0.01	2.5	3.76	0.14	0.00	0.46

a Data are expressed as F values, and *, **, ***, **** indicate P<0.05, 0.01, 0.001 and 0.0001, respectively. Abbreviations are: FW- fresh weight; Temp- Temperature; N- Nitrogen; 3-Way- CO_2_×Temperature**×**Nitrogen.

## Discussion

### Variation in Fruit Number, Weight and Yield of Strawberry

Fruit yield of strawberry per plant is composed of fruit dry weight (FDW) and fruit number (FN), while FDW is affected by the total achene number (TAN) and dry matter accumulated per achene (DMA), since achenes (actual seeds) are considered to be involved in regulating strawberry fruit development [27]. Therefore, the treatment effects on either FDW or FN will highlight the effect on fruit yield under those treatments.

**Figure 4:**
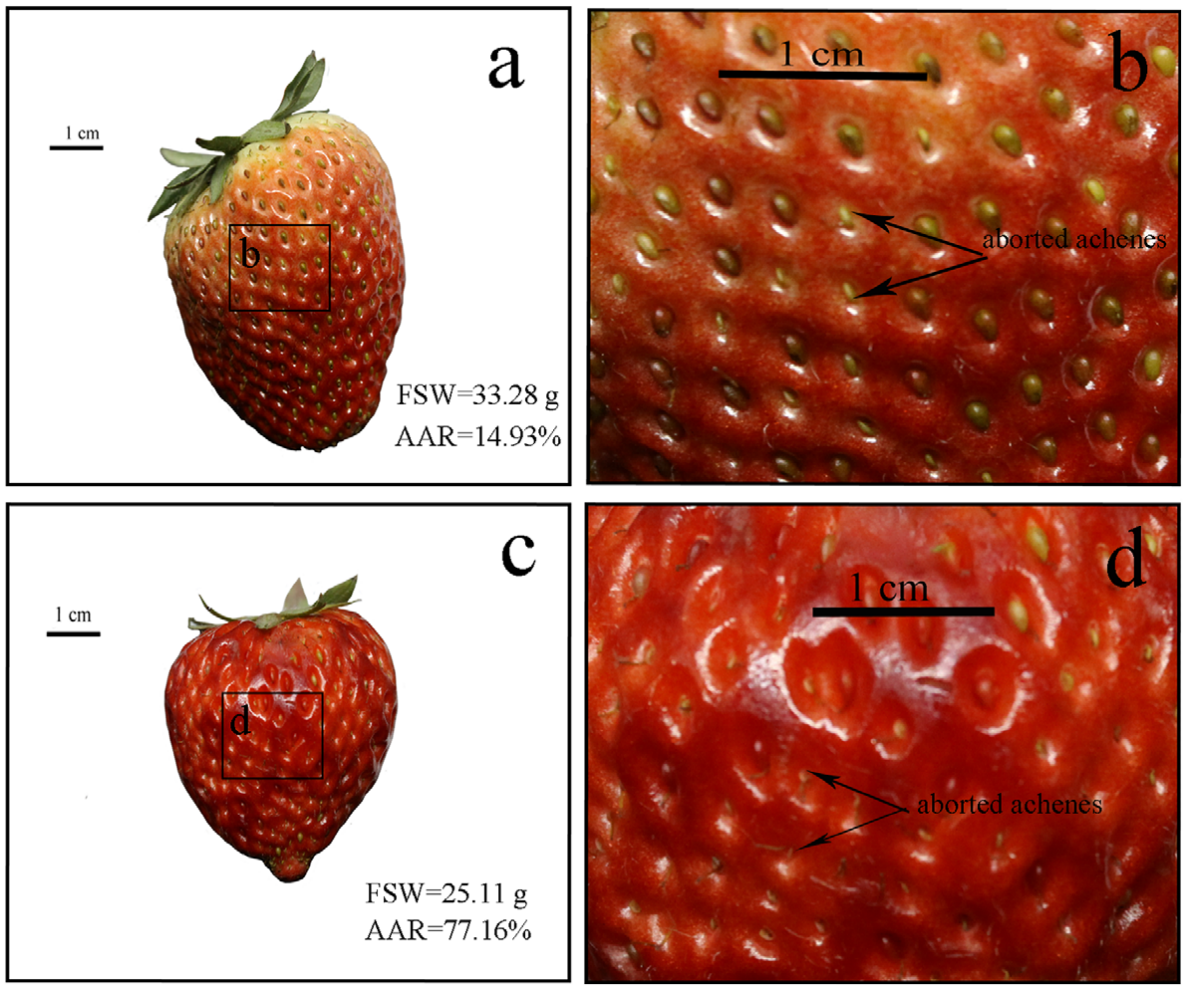
Strawberry fruits with similar fruit fresh weight (FSW) but different achene abortion rates (AAR). (a) strawberry fruits with low achene abortion rate; (b) part of the figure a is amplified to indicate the aborted achenes; (c) strawberry fruit with high achene abortion rate; (d) part of the figure c is amplified to indicate the aborted achenes.

Compared to the corresponding treatments in ambient CO_2_ (T and TN treatments), elevated CO_2_ further reduced the fruit yield at high temperature (CT and CTN treatments). Yield reductions, which were further enhanced by elevated CO_2_ at high temperature during flowering and fruit development, also have been documented in other crops such as rice, wheat, grain sorghum, kidney bean, dry bean, soybean, peanut and tomato, though the extreme temperatures were much higher than the one used in this study [7], [9]–[10], [28]–[31]. Commonly, the increased seed abortion caused by decreased pollen production [30], lower pollen reception by stigma due to anther indehiscence [32], and lower pollen viability due to degeneration of tapetum layer and decreased carbohydrate metabolism [33]–[35] during flower development and opening, resulted in the reduction of crop yield at high temperature. The exact mechanism of the increased susceptibility of these processes to high temperature at elevated CO_2_ is still unclear, but the small increase in tissue temperatures (owing to decreased leaf conductance) which reduces the ceiling temperatures for seed-set by about 2°C is one possible explanation [9]. However, in this study, achene abortion (as seed abortion in other crops) caused by the negative impacts of warmer tissue temperatures on flower development and opening were insufficient to explain the reduction of strawberry yield under high CO_2_ concentration. Nitsch [36] reported that fruit production of strawberry was proportional to the extent of achene fertilization, and strawberry fruit size was positively related to the number of fertilized achenes. Thus, if the yield reduction at high temperature and elevated CO_2_ was caused by achene abortion, the correlation between FDW and total number of achenes (TAN) will be lower than the correlation between FDW and total number of fertilized achenes (TFA), because aborted achenes will decrease the accumulation of dry matter. However, the correlation between FDW and TFA was 28% lower than the correlation between FDW and TAN, which suggested that the adverse effects of elevated CO_2_ and high temperature on achene fertilization were not the major causes of yield reduction. In other words, TAN, rather than TFA, strongly correlating to FDW implied that a possible regulation mechanism existed in strawberry fruit responding to the occurrence of achene abortion. When achene abortion occurs, the remnant fertile achenes may be stimulated to increase the capacity of dry matter accumulation to offset the reduction of aborted achenes. The observation in Figure 4 indicated that even great achene abortion rates occurred the fruits with similar fruit size generally had the similar fruit weight, which also confirmed achene abortion having rather limited effect on FDW and then yield reduction. In addition, the slopes of linear regression which indicated the dry matter accumulated per achene (DMA) increased at elevated CO_2_ and low nitrogen, but have no benefit in maintaining fruit yield at high temperature and elevated CO_2_ when yield reduction occurred. Therefore, we propose that achene abortion was not the main cause of yield reduction, and the increased DMA at elevated CO_2_ also has no benefit in maintaining fruit yield at high temperature. Indeed, TAN, which was determined by flower induction, affected the change of FDW and contributed greatly to the reduction of fruit yield at elevated CO_2_ and high temperature.

Besides TAN, the inflorescences of strawberry were hand-pollinated to keep to a minimum inflorescence abortion, thus FN was also determined by flower induction. Therefore, as an alternative explanation, the reduction of strawberry yield in CT and CTN treatments was mainly caused by the inhibition of flower induction, which could be suppressed by high temperature and other environment factors [37]. In other words, the fewer number of inflorescences and the smaller umbel size of strawberry during flower induction resulted in the reduction of fruit yield at elevated CO_2_ and high temperature. Low temperature, as one of many environment factors, can affect flower induction in plant through many physiological pathways, including vernalization and gibberellin (GA) biosynthesis [37]. Exposure to the prolonged cold of winter, through this process called vernalization, is required to permit flowering of June-bearing strawberry plants [38]–[39]. However, the promotion of flowering by vernalization could be reduced or even completely suppressed by high temperature [37], and devernalization by high day temperature over-riding the effect of low night temperature which induced flower bud initiation has been found in some cultivars of strawberry [40]. In this study, the warmer tissue temperatures in day time at elevated CO_2_ may cause devernalization of the plants, and eventually inhibit flower induction. Besides the vernalization pathway, GA biosynthesis and signaling, including genes such as *GA*_1_, *GAI*, *RGA*, *FPF*_1_ and *AtMYB*_33_, plays an important role in flower induction in *Arabidopsis* [37]. It was suggested that temperature effect may be mediated by changes in the level of active endogenous GA_s_ [41]. Su et al. [42]reported that the flowering shoots of *Phalaenopsis hybrida* grown under high temperature contained lower levels of GA_1_, GA_19_, GA_20_ and GA_53_ than GA_3_-treated and cold-induced plants. They also found relatively low level of GA_1_ and high level of GA_8_ in shoot-tips of warm control (non-flowering) plants compared to plants whose flowering was promoted with GA_3_ or cool-temperatures. Tayor et al. [43] studied the possible role of endogenous GAs in the control of flowering in strawberry and identified eight 13-hydroxylated GAs from leaf tissues of the short-day cv. Elasnta. Thus, the change of endogenous GAs biosynthesis induced by warmer tissue temperatures may be another reason of inhibition of flower induction at elevated CO_2_.

Surprisingly, nitrogen application further decreased fruit yield in CTN treatment than in CT treatment. Though, nitrogen application greatly improved fruit size, the FN level at high temperature was reduced to the extent of nearly a half of low nitrogen treatments. This reduction implied that the induction of inflorescences was greatly reduced at high nitrogen, but umbel size increased. As reported, increase of mineral supply to the roots delayed flowering in several mutants of the photoperiod and autonomous pathways, as well as in wild-type plants in *Arabidopsis* [44]. An important part of this inhibition was presumably due to nitrogen [45]. Corbesier et al. [46] reported high nitrate-supplement reduced the export of sucrose towards the shoot apical meristem at floral transition, and the decrease of FN in high nitrogen treatment in this study is probably caused due to this reason.

Our study suggests that, as a fruit tree, strawberry is idiographic with a highly sensitive requirement of a narrow range of temperature for flower induction. We propose that reduced flower induction plays an important role in the reduction of strawberry yield at high temperature and elevated CO_2_. Further, the adverse effect of high temperature and elevated CO_2_ on fruit yield were not ameliorated but rather exacerbated under high nitrogen condition. However, nitrogen supply did improve fruit quality by increasing the fruit weight.

### Variation in Taste- and Health -related Compounds

The increased dry matter-content (DMC) of the fruits was probably due to the increased non-structural carbohydrates sourced from the increased net photosynthetic rate of strawberry at elevated CO_2_ [47]–[48]. The non-structural carbohydrates including fructose (the dominant sugar), glucose and sucrose, contribute directly to the perceived sweetness of the fruit, and these sugars account for more than 990 g kg^−1^ of the total sugars in ripe strawberries [49]. Therefore, elevated CO_2_ which increased fructose, glucose and total sugar levels relative to other taste related compounds would improve the perception of fruit sweetness.

At elevated CO_2_, decrease in tissue nitrogen content has been widely reported, but there was significant variation in different taxa [50]. In this study, fruit nitrogen content (FNC) decreased nearly 11% at elevated CO_2_ and this value was in the range of the reduction of seed nitrogen content (15%) at elevated CO_2_ [15]. Dilution hypothesis suggests that the decrease in tissue nitrogen content under elevated CO_2_ results from the dilution due to accumulation of non-structural carbohydrates or plant secondary compounds [51]. In this study, the decline in FNC at elevated CO_2_ may be caused by dilution effect of accumulated non-structural carbohydrates, since elevated CO_2_ greatly increased leaf photosynthesis and accelerated the accumulation of these compounds. At elevated CO_2_, the total antioxidant capacity and all antioxidant compounds in strawberry fruits decreased nearly 27.5% (from 19% to 37%) at low nitrogen and 9.5% (from 3% to 13%) at high nitrogen, whilst DMC increased 23.7% and 12.5% in the corresponding treatments, respectively. The increase of DMC was proportional to the decrease in total antioxidant capacity and all antioxidant compounds at elevated CO_2_, which implied that the reduction of total antioxidant capacity and all antioxidant compounds in strawberry fruits were mainly caused by the dilution effect of accumulated non-structural carbohydrates, though the dilution effect on individual antioxidant compounds varied.

The extent of decrease in total antioxidant capacity and antioxidant compounds was greater at low nitrogen than at high nitrogen, implying that nitrogen application greatly modified the treatment effect of elevated CO_2_ on these compounds. From the results, the greater decrease of antioxidant activity at low nitrogen mainly came from the higher antioxidant levels in ambient CO_2_ and lower antioxidant levels at elevated CO_2_, when compared to these compounds at high nitrogen. Commonly, the change of antioxidant activity results from the change of ROS, and these antioxidant compounds were evolved to protect plants from oxidative damage [52]. It is known that environment stresses, including nitrogen starvation [25], may increase the production of ROS. We have summarized four possible causes of the increase of ROS under nitrogen deficiency including: (1) “physiological trade-off” between plant growth and secondary metabolite production in GDB framework [25]–[26], [53]; (2) accelerated senescence of plant tissues or organs [54]–[55]; (3) limitation of CO_2_ uptake efficiency and accumulation of reducing power due to accumulation of H_2_O_2_ in nitrogen deficient plants, which is known to decrease stomatal opening [56]–[57]; (4) surplus electron flow leading to enhanced oxygen photo-reduction in the chloroplast via the Mehler reaction as the ratio of Rubisco activity declined under nitrogen deficiency [58].

In this study, the increased antioxidant activity in strawberry fruit at low CO_2_ concentration and low nitrogen treatments could not be explained satisfactorily with the reasons mentioned above except the first one. Obviously, reason 3 and 4 were not suitably explained in fruit, while reason 2 contrasted with recent research that elevated CO_2_ accelerated senescence of plant tissues or organs and would increase antioxidant level in them [59]–[61]. Therefore, reason 1 will be a possible explanation that secondary metabolites such as phenolics are accumulated at low nitrogen [25]. Meanwhile, ROS which is involved in the signaling and perception of nitrogen deficiency is also increased [26]. The antioxidant levels decreased in CT and C treatments (though the extent was rather small) suggesting that the effect of nitrogen deficiency on antioxidant level has been modified by the elevated CO_2_. We speculate that the reduced FNC in these treatments may inhibit the activity and amount of relevant enzymes involved in perception of nitrogen deficiency and synthesis of secondary metabolites, and negatively affect the antioxidant levels.

### Conclusions

Overall, our study illustrates the combined effects of elevated CO_2_, nitrogen and temperature on strawberry yield and quality. At low temperature, elevated CO_2_ greatly improved the fruit yield by increasing fruit number and fruit weight. However, at high temperature, elevated CO_2_ decreased fruit yield. This decrease was mainly caused by the fewer induced inflorescences and smaller induced umbel size which eventually reduced fruit number and fruit weight, respectively. Moreover, elevated CO_2_ increased the levels of dry matter-content, fructose, glucose, total sugar and sweetness index per dry matter, but decreased fruit nitrogen content, total antioxidant capacity and all antioxidant compounds per dry matter in strawberry fruit. The reduction of fruit nitrogen content and antioxidant activity was mainly caused by the dilution effect of accumulated non-structural carbohydrates sourced from the increased net photosynthetic rate during fruit development. Thus, the quality of strawberry fruit would increase because of the increased sweetness and the similar amount of fruit nitrogen content, DPPH, ABTS and all antioxidant compounds per fresh matter at elevated CO_2_. Interestingly, nitrogen application had no beneficial effect on the fruit yield, but greatly increased fruit weight among all treatments. Fruit quality such as antioxidant activity increased at high nitrogen and elevated CO_2_, but decreased at high nitrogen and low CO_2_. Considering all treatment effects, we conclude that elevated CO_2_ improved the production of strawberry (including yield and quality) at low temperature, but decreased it at high temperature. In addition, the dramatic fluctuation in strawberry yield between low and high temperature at elevated CO_2_ implies that more attention should be paid to the process of flower induction under climate change especially in fruits that require winter chilling for reproductive growth, as chronic and steady reduction in winter chill is expected [62]. Therefore, efforts should be made to develop cultivars that require less winter chill for future climate.

## Materials and Methods

### Plant Material and Experimental Design

Four large growth chambers with an internal chamber height of 2.20 m and a growth area of 1.0 m^2^ were used for the experiment. All chambers have air temperature, relative humidity and carbon dioxide control. Photosynthetic active radiation (PAR) was about 600 µmol m^−2^ s^−1^, and relative humidity was controlled at 80% by an air humidifier 24 hours a day. CO_2_ was injected automatically into the chambers all day and night, and its concentration was controlled using a CO_2_ delivery system and chamber vents. An individual LICOR infrared gas analyzer (LI-800 GasHound CO_2_ Analyzer, LI-COR, Nebraska, USA) was used to monitor the CO_2_ levels for each chamber independently, and the accuracy of the analyzer was ±2%.

The experimental design consisted of a three-way randomized block with four replications. The treatments consisted of two day/night temperature levels [20/15°C (T_A_), 25/20°C (T_A_ +5°C)], two CO_2_ concentrations [360 and 720 µmol CO_2_ mol^−1^ air], and two nitrogen application levels [0% (distilled water) and 0.01% NH_4_NO_3_]. The temperature and CO_2_ treatments were randomly allocated in each of the four growth chambers as follows:

- Chamber 1-T_A_ +5°C and 360 µmol CO_2_ mol^−1^,
- Chamber 2-T_A_ +5°C and 720 µmol CO_2_ mol^−1^,
- Chamber 3-T_A_ and 360 µmol CO_2_ mol^−1^,
- Chamber 4-T_A_ and 720 µmol CO_2_ mol^−1^.

Fifty milliliter of 0.01% NH_4_NO_3_ solution was applied twice a week per plant at the beginning of 1 December 2010 and lasted for nearly 6 months. A fixed day length of 10 h from 7∶00 AM to 17∶00 PM, which corresponds to the day length of early spring in Zhejiang, was used.

The strawberry cultivar used in this study was Toyonoka (*Fragaria* **×** *ananassa* Duch. cv. Toyonoka) a short-day cultivar which need short-day and low temperature (chilling) treatments to accelerate flower bud initiation [63]–[64], and now is widely planted in Zhejiang. Strawberry seedlings were planted in 25 cm**×**18 cm pots using field soil (red soil, total nitrogen content 0.96 g/kg dry soil). Prior to the treatments in chambers, plants grew under the ambient autumn temperatures of Jinhua, Zhejiang, in an unheated greenhouse from November to December for one month (chilling and short-day treatments), and the mean daily temperature in November was about 13.2°C. All plants were watered daily and fertilized weekly with 150 ml per plant of Peters fertilizer (20∶20:20, N/P/K). Plants with similar height and crown diameter were moved to chambers and 8 pots were placed in each chamber and four pots per treatment. The plants in each chamber were rotated inside chambers per week and between chambers per month to reduce the microclimate effects of different chambers. Blossoms were self-pollinated by hand using a small brush. As daily routine, the ripeness of fruit was determined by color, and firm red-ripe fruits free from defects or decay were harvested from each growth chamber during the fruiting stage. Fruit dry weight, fruit number, total achenes and total aborted achenes were determined. All of berries were graded in three size classes (grade 1<0.4 g; grade 2, 0.4–0.7 g; grade 3>0.7 g) according to FDW. The berries of each plant were cut into small slices, mixed, and frozen at −24°C for analyzing until the end of the harvest season.

### Fruit Sample Preparation

To prepare the fruit samples, four 100 g samples of berries from four replicates of each treatment were homogenized for 2 min in a rotating blade homogenizer (Midea, JP351, China). Solution of homogenate extract (2 g) in methanol (25 ml) was used for determination of total flavonoid, total phenolic, DPPH and ABTS. Solution of homogenate extract (2 g) in distilled water (25 ml) was used for determination of anthocyanin content. All compounds mentioned above in each sample from each plant were measured in triplicate and four samples of each treatment were determined.

### Determination of Antioxidant Compounds Content

The amount of all the antioxidant compounds was determined according to Zheng et al. and Lu et al. [65]–[68]. The total flavonoid content was determined by a colorimetric assay with modifications. Briefly, 0.5 mL extract solution was separately mixed with 1.5 mL of methanol, 0.1 mL of 2% aluminum chloride, 0.1 mL of 1 M potassium acetate, and 2.8 mL of distilled water, and left at room temperature for 30 min. The absorbance of the reaction mixture was measured at 415 nm using a UV-vis spectrophotometer (Jinghua, JH752, China). The total flavonoid content was expressed as rutin equivalents in milligrams per gram dry weight of strawberry.

The total phenolic content was determined colorimetrically using Folin–Ciocalteau reagent, with modifications. The total phenolic assay was conducted by mixing 8.25 mL of deionized water, 0.5 mL of extract, 0.75 mL of 20% Na_2_CO_3_, and 0.5 mL of Folin–Ciocalteu reagent. After 40 min of reaction in a water bath at 40°C, the absorbance at 755 nm was measured using a spectrophotometer. Results were expressed as gallic acid equivalents milligrams per gram of dry weight of strawberry.

The total anthocyanins content was determined with a modified pH differential method, using two buffer systems: potassium chloride 0.025 M at pH 1.0 and sodium acetate 0.4 M at pH 4.5. Briefly, 1 mL of sample was transferred to a 10 mL volumetric flask and made up with each buffer. The absorbance of each equilibrated solution was then measured at 510 and 700 nm, using a UV-vis spectrophotometer. Quartz cuvettes of 1 cm path length were used, and all measurements were carried out at room temperature (25°C). Absorbance readings were made against distilled water as a blank. The total anthocyanins content was calculated on the basis of cyanidin-3-glucoside with a molecular weight of 445.2 g/mol and an extinction coefficient of 29600 L/mol · cm, as
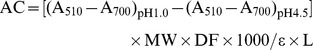
(1)Where MW is the molecular weight of cyanidin-3-glucoside, DF is the dilution factor, L is the path length in cm, and ε is the molar extinction coefficient for cyanidin-3-glucoside. Results were expressed as milligram cyanidin-3-glucoside equivalents per gram of dry weight of strawberry.

### Determination of Total Antioxidant Capacity (DPPH and ABTS)

The DPPH free radical scavenging activity was evaluated according to the method of our previous study [65]–[66]. The extracts (0.1 mL) of strawberry in ethanol were reacted with 10 mL of 0.03 g/L DPPH ethanol solution at room temperature. The extract (0.1 mL) with 10 mL distilled water was used as control. The absorbance was measured at 517 nm after 30 min of reaction in the dark. DPPH radical scavenging capacity was expressed as Trolox equivalent antioxidant capacity (µmol of Trolox/1 g of dry strawberry fruits).

The ABTS assay was based on the method of Re et al. [69] with slight modification. ABTS^•+^ reagent was produced by reacting 10 mL of 7 mM ABTS solution with 178 µL of 140 mM potassium persulfate aqueous in the dark at room temperature for 13 h before use. The ABTS^•+^ solution was diluted with ethanol to appropriate absorbance. One-tenth of a milliliter of extract was added to 3.9 mL of diluted ABTS^•+^ solution to react in the dark at room temperature for 6 min, and the absorbance at 732 nm was recorded. Trolox was used as standard with the final concentration ranging from 0 to 16.5 µM. Results were expressed as Trolox equivalent antioxidant capacity (µmol of Trolox/1 g of dry strawberry fruits).

### Determination of Fruit Nitrogen Content

Fruit nitrogen content was determined by mico-Kjeldahl digestion method [70], with modifications. Briefly, 0.5 g dry fine powder of strawberry fruit was accurately weighted into mico-Kjedlahl flasks to which the catalyst mixture (0.3% TiO_2_, 0.3% CuSO_4_, and 10% K_2_SO_4_ on a weight basis) and concentrated sulfuric acid (10 mL) were added. The digests were heated for 1.5 h beyond the point when the solutions had cleared. They were then cooled and diluted to 50 mL with distilled water. After addition of 3 ml of 20 g/L H_3_BO_3_ solution in the inner chamber of a clean Conway dish, 4 mL diluted digest was added in the outer chamber. The covered Conway dishes were sealed and incubated at 40°C for 24 h. The absorbed ammonia in H_3_BO_3_ solution was titrated with 0.02 mol/L HCl solution. The results were expressed as milligram per gram of dry weight of strawberry. Each sample from each plant was measured in triplicate and four samples of each treatment were determined.

### Analysis of Sugars Using HPLC

For analysis of sugars, 10 g of snap-frozen strawberry powder (wet) were stirred by a magnetic stirring apparatus in 100 ml of extraction solution containing 90 ml of distilled water, 5 ml of 1 mol l^−1^ zinc acetate and 5 ml of 0.25 mol l^−1^ potassium ferrocyanide for 30 min at room temperature. The solution was filtered through a membrane-filtered supernatant (Ø 0.26 µm). Glucose, fructose and sucrose were analyzed by injection of a 50 µl sample volume into a DuoFlow HPLC system (Bio-RAD, USA) using a Sepax Amethyst-Amino column, 250 mm**×**4.6 mm diameter, 5 µm particle size (Sepax, USA; Part no. 322305-4625). The column temperature of 20°C was controlled and an acetonitrile: pure water solution (80∶20 v/v) was used as mobile phase (flow rate 0.8 ml min^−1^). Carbohydrates were detected with a refractive index detector (RID-10A, Japan) and their concentrations were calculated by comparing sample peak area to standards using OriginPro 8.5 software. Each sample from each plant was measured in triplicate and four samples of each treatment were determined. The results were recalculated per dry mass.

The sweetness index was calculated by multiplying the sweetness coefficient of each individual sugar (glucose = 1, fructose = 2.3 and sucrose = 1.35), as described by Keutgen and Pawelzik [71].

### Statistical Analyses

Data in this study were subjected to analysis of variance, and means were compared by least significant difference (LSD). Multivariate general linear model function (MGLM) was performed to analyze the main effects of CO_2_ concentration, air temperature and nitrogen input combined with their interactions on the quality of strawberry growing in chambers. Regression analysis was conducted to examine relationships between fruit dry weight and total achene number. In this study, all statistical analyses were conducted using SAS software (SAS Institute Inc., Cary, NC, USA).

## References

[1] IPCC (2007). Climate change 2007: The physical science basis. Contribution of working group I to the fourth assessment report of the intergovernmental panel on climate change.. Cambridge: Cambridge University Press.

[2] Xiong W, Lin E, Ju H, Xu Y (2007). Climate change and critical thresholds in China’s food security.. Climatic Change.

[3] Santos BM, Chandler CK (2009). Influence of nitrogen fertilization rates on the performance of strawberry cultivars.. Int J Fruit Sci.

[4] Konsin M, Voipio I, Palonen P (2001). Influence of photoperiod and duration of the short-day treatment on vegetative growth and flowering of strawberry (*Fragaria*×*ananassa* Duch.).. J Hortic Sci Biotech.

[5] Kumakura H, Shishido Y (1995). Effects of temperature and light conditions on flower initiation and fruit development in strawberry.. Jpn Agr Res Q.

[6] Chen K, Hu GQ, Lenz F (1997). Effect of CO_2_ concentration on strawberry. IV. Carbohydrate production and accumulation.. J Appl Bot-Angew Bot.

[7] Matsui T, Namuco OS, Ziska LH, Horie T (1997). Effects of high temperature and CO_2_ concentration on spikelet sterility in indica rice.. Field Crop Res.

[8] Baker JT, Allen LH, Boote KJ, Jones P, Jones JW (1989). Response of soybean to air temperature and carbon dioxide concentration.. Crop Sci.

[9] Prasad PVV, Boote KJ, Allen LH, Thomas JMG (2002). Effects of elevated temperature and carbon dioxide on seed-set and yield of kidney bean (*Phaseolus vulgaris* L.).. Global Change Biol.

[10] Prasad PVV, Boote KJ, Allen LH, Thomas JMG (2003). Super-optimal temperatures are detrimental to peanut (*Arachis hypogaea* L.) reproductive processes and yield under both ambient and elevated carbon dioxide.. Global Change Biol.

[11] Ahmed FE, Hall AE, Madore MA (1993). Interactive effects of high temperature and elevated carbon dioxide concentration on cowpea [*Vigna unguiculata* (L.) Walp.].. Plant Cell Environ.

[12] Wheeler TR, Batts GR, Ellis RH, Hadley P, Morison JIL (1996). Growth and yield of winter wheat (*Triticum aestivum*) crops in response to CO_2_ and temperature.. J Agr Sci.

[13] Reddy KR, Hodges HF, Kimball BA, Reddy KR, Hodges HF (2000). Crop ecosystem responses to climate change: cotton..

[14] Aerts R, Chapin FS (2000). The mineral nutrition of wild plants revisited: a re-evaluation of processes and patterns.. Adv Ecol Res.

[15] Hikosaka K, Kinugasa T, Oikawa S, Onoda Y, Hirose T (2011). Effects of elevated CO_2_ concentration on seed production in C_3_ annual plants.. J Exp Bot.

[16] Li W, Han X, Zhang Y, Li Z (2007). Effects of elevated CO_2_ concentration, irrigation and nitrogenous fertilizer application on the growth and yield of spring wheat in semi-arid areas.. Agr Water Manage.

[17] Xiao G, Zhang Q, Wang R, Xiong Y (2009). Effects of elevated CO_2_ concentration, supplemental irrigation and nitrogenous fertilizer application on rain-fed spring wheat yield.. Acta Ecol Sin.

[18] Yoshida H, Horie T, Nakazono K, Ohno H, Nakagawa H (2011). Simulation of the effects of genotype and N availability on rice growth and yield response to an elevated atmospheric CO_2_ concentration.. Field Crop Res.

[19] Deng X, Woodward FI (1998). The growth and yield responses of *Fragaria* × *ananassa* to elevated CO_2_ and N supply.. Ann Bot-London.

[20] Heinonen IM, Meyer AS, Frankel EN (1998). Antioxidant activity of berry phenolics on human low-density lipoprotein and liposome oxidation.. J Agric Food Chem.

[21] Wang SY, Bunce JA, Maas JL (2003). Elevated carbon dioxide increases contents of antioxidant compounds in field-grown strawberries.. J Agric Food Chem.

[22] Caldwell CR, Britz SJ, Mirecki RM (2005). Effect of temperature, elevated carbon dioxide, and drought during seed development on the isoflavone content of dwarf soybean [*Glycine max* (L.) Merrill] grown in controlled environments.. J Agric Food Chem.

[23] Gonçalves B, Falco V, Moutinho-Pereira J, Bacelar E, Peixoto F (2009). Effects of elevated CO_2_ on grapevine (*Vitis vinifera* L.): volatile composition, phenolic content, and in vitro antioxidant activity of red wine.. J Agric Food Chem.

[24] Levine LH, Paré PW (2009). Antioxidant capacity reduced in scallions grown under elevated CO_2_ independent of assayed light intensity.. Adv Space Res.

[25] Kováčik J, Bačkor M (2007). Changes of phenolic metabolism and oxidative status in nitrogen-deficient *Matricaria chamomilla* plants.. Plant Soil.

[26] Dixon RA, Harrison MJ, Lamb CJ (1994). Early events in the activation of plant defense responses.. Annu Rev Phytopathol.

[27] Quesada MA, Blanco-Portales R, Posé S, García-Gago JA, Jiménez-Bermúdez S (2009). Antisense down-regulation of the *FaPG1* gene reveals an unexpected central role for polygalacturonase in strawberry fruit softening.. Plant Physiol.

[28] Saini HS, Aspinall D (1982). Abnormal sporogenesis in wheat (*Triticum aestivum* L.) induced by short periods of high temperature.. Ann Bot-London.

[29] Sato S, Peet MM, Thomas JF (2000). Physiological factors limit fruit set of tomato (*Lycopersicon esculentum* Mill.) under chronic, mild heat stress.. Plant Cell Environ.

[30] Prasad PVV, Boote KJ, Allen LH (2006). Seed-set, seed yield and harvest index of grain-sorghum [*Sorghum bicolor* (L.) Moench] are more severe at elevated carbon dioxide due to higher tissue temperatures.. Agr Forest Meteorol.

[31] Prasad PVV, Boote KJ, Allen LH, Sheehy JE, Thomas JMG (2006). Species, ecotype and cultivar differences in spikelet fertility and harvest index of rice in response to high temperature stress.. Field Crop Res.

[32] Porch TG, Jahn M (2001). Effects of high-temperature stress on microsporogenesis in heat-sensitive and heat-tolerant genotypes of *Phaseolus vulgaris*.. Plant Cell Environ.

[33] Datta R, Chourey PS, Pring DR, Tang HV (2001). Gene-expression analysis of sucrose-starch metabolism during pollen maturation in cytoplasmic male-sterile and fertile lines of sorghum.. Sex Plant Reprod.

[34] Suzuki K, Tsukaguchi T, Takeda H, Egawa Y (2001). Decrease of pollen stainability of green bean at high temperatures and relationship to heat tolerance.. J Am Soc Hortic Sci.

[35] Pressman E, Peet MM, Pharr DM (2002). The effect of heat stress on tomato pollen characteristics is associated with changes in carbohydrate concentration in the developing anthers.. Ann Bot-London.

[36] Nitsch JP (1952). Plant hormones in the development of fruits.. Q Rev Biol.

[37] Bernier G, Périlleux C (2005). A physiological overview of the genetics of flowering time control.. Plant Biotechnol J.

[38] Sung S, Amasino RM (2004). Vernalization in *Arabidopsis thaliana* is mediated by the PHD finger protein VIN3.. Nature.

[39] Avigdori-Avidov H, Goldschmidt EE, Kedar N (1977). Involvement of endogenous gibberellins in the chilling requirements of strawberry (*Fragaria* × *ananassa* Duch.).. Ann Bot-London.

[40] Yamasaki A, Tanaka K, Yoshida M, Miura H (2000). Effects of day and night temperature on flower-bud formation and bolting of Japanese bunching onion (*Allium fistulosum* L.).. J Jpn Soc Hortic Sci.

[41] Grindal G, Junttila O, Reid JB, Moe M (1998). The response to gibberellin in *Pisum sativum* grown under alternating day and night temperature.. J Plant Growth Regul.

[42] Su WR, Chen WS, Koshioka M, Mander LN, Hung LS (2001). Changes in gibberellin levels in the flowering shoot of *Phalaenopsis hybrida* under high temperature conditions when flower development is blocked.. Plant Physiol Bioch.

[43] Taylor DR, Blake PS, Browning G (1994). Identification of gibberellins in leaf tissues of strawberry (*Fragaria* × *ananassa* Duch.) grown under different photoperiods.. Plant Growth Regul.

[44] van Tienderen PH, Hammad I, Zwaal FC (1996). Pleiotropic effects of flowering time genes in the annual crucifer *Arabidopsis thaliana* (Brassicaceae).. Am J Bot.

[45] Bernier G, Kinet JM, Sachs RM (1981). The physiology of flowering, Vol. I. Boca Raton: CRC Press.. 149 p.

[46] Corbesier L, Vincent C, Jang S, Fornara F, Fan Q (2007). FT protein movement contributes to long-distance signaling in floral induction of *Arabidopsis*.. Science.

[47] Lieten F (1997). Effect of CO_2_ enrichment on greenhouse grown strawberry.. Acta Hort.

[48] Chen K, Hu GQ, Lenz F (1997). Effects of CO_2_ concentration on strawberry. VI. Fruit yield and quality.. J Appl Bot-Angew Bot.

[49] Wang SY, Bunce JA (2004). Elevated carbon dioxide affects fruit flavor in field-grown strawberries (*Fragaria* × *ananassa* Duch).. J Sci Food Agr.

[50] Jablonski LM, Wang X, Curtis PS (2002). Plant reproduction under elevated CO_2_ conditions: a meta-analysis of reports on 79 crop and wild species.. New Phytol.

[51] Taub DR, Wang X (2008). Why are nitrogen concentrations in plant tissues lower under elevated CO_2_? A critical examination of the hypotheses.. J Integr Plant Biol.

[52] Giorgi A, Mingozzi M, Madeo M, Speranza G, Cocucci M (2009). Effect of nitrogen starvation on the phenolic metabolism and antioxidant properties of yarrow (*Achillea collina* Becker ex Rchb.).. Food Chem.

[53] Herms DA, Mattson WJ (1992). The dilemma of plants: to grow or defend.. Q Rev Biol.

[54] Casano LM, Martin M, Sabater B (1994). Sensitivity of superoxide dismutase transcript levels and activities of oxidative stress is lower in mature-senescent than in young barley leaves.. Plant Physiol.

[55] Buchanan-Wollaston V, Earl S, Harrison E, Mathas E, Navabpour S (2003). The molecular analysis of leaf senescence–a genomics approach.. Plant Biotechnol J.

[56] Grossman A, Takahashi H (2001). Macronutrient utilization by photosynthetic eukaryotes and the fabric of interactions.. Annu Rev Plant Physiol Plant Mol Biol.

[57] Neill S, Desikan R, Hancock J (2002). Hydrogen peroxide signaling.. Curr Opin Plant Biol.

[58] Lin YL, Chao YY, Huang WD, Kao CH (2011). Effect of nitrogen deficiency on antioxidant status and Cd toxicity in rice seedlings.. J Plant Growth Regul.

[59] Zhu C, Zhu J, Zeng Q, Liu G, Xie Z (2009). Elevated CO_2_ accelerates flag leaf senescence in wheat due to ear photosynthesis which causes greater ear nitrogen sink capacity and ear carbon sink limitation.. Funct Plant Biol 36.

[60] Franzaring J, Weller S, Schmid I, Fangmeier A (2011). Growth, senescence and water use efficiency of spring oilseed rape (*Brassica napus* L. cv. Mozart) grown in a factorial combination of nitrogen supply and elevated CO_2_.. Environ Exp Bot.

[61] McConnaughay KDM, Bassow SL, Berntson GM, Bazzaz FA (1996). Leaf senescence and decline of end-of-season gas exchange in five temperate deciduous tree species grown in elevated CO_2_ concentrations.. Global Change Biol.

[62] Baldocchi D, Wong S (2007). Accumulated winter chill is decreasing in the fruit growing regions of California.. Climatic Change.

[63] Ledesma NA, Nakata M, Sugiyama N (2008). Effect of high temperature stress on the reproductive growth of strawberry cvs. ‘Nyoho’ and ‘Toyonoka’.. Sci Hortic-Amsterdam.

[64] Morishita M, Mochizuki T, Yamakawa O (1993). Flower induction and selection on earliness of strawberry seedlings by short-day and low night temperature treatment.. J Jpn Soc Hortic Sci.

[65] Zheng H, Jiang L, Lou H, Hu Y, Kong X (2011). Application of artificial neural network (ANN) and partial least-squares regression (PLSR) to predict the changes of anthocyanins, ascorbic acid, total phenols, flavonoids, and antioxidant activity during storage of red bayberry juice.. J Agric Food Chem.

[66] Zheng H, Lu H, Zheng Y, Lou H, Chen C (2010). Automatic sorting of Chinese jujube (*Zizyphus jujube* Mill. cv. ‘hongxing’) using chlorophyll fluorescence and support vector machine.. J Food Eng.

[67] Lu H, Zheng H, Hu Y, Lou H, Kong X (2011). Bruise detection on red bayberry (*Myrica rubra* Sieb. & Zucc.) using fractal analysis and support vector machine.. J Food Eng.

[68] Lu H, Zheng H, Lou H, Jiang L, Chen Y (2010). Using neural networks to estimate the losses of ascorbic acid, total phenols, flavonoid, and antioxidant activity in asparagus during thermal treatments.. J Agric Food Chem.

[69] Re R, Pellegrini N, Proteggente A, Pannala A, Yang M (1999). Antioxidant activity applying an improved ABTS radical cation decolorization assay.. Free Radical Bio Med.

[70] American Society for Testing Materials (2002). Standard test method for total Kjeldahl nitrogen in water.. West Conshohocken: ASTM International.

[71] Keutgen A, Pawelzik E (2007). Modifications of taste-relevant compounds in strawberry fruit under NaCl salinity.. Food Chem.

